# tRF-3013b inhibits gallbladder cancer proliferation by targeting TPRG1L

**DOI:** 10.1186/s11658-022-00398-6

**Published:** 2022-11-18

**Authors:** Lu Zou, Yang Yang, Biyu Zhou, Weijian Li, Ke Liu, Guoqiang Li, Huijie Miao, Xiaoling Song, Jiahua Yang, Yajun Geng, Maolan Li, Runfa Bao, Yingbin Liu

**Affiliations:** 1 Department of Biliary-Pancreatic Surgery, Renji Hospital, School of Medicine, Shanghai Jiaotong University, Shanghai, 200127 China; 2 Shanghai Key Laboratory of Biliary Tract Disease Research, Shanghai, 200092 China; 3 Shanghai Cancer Institute, Renji Hospital, Shanghai Jiaotong University School of Medicine, Shanghai, 200032 China; 4 Department of Plastic and Reconstructive Surgery, Renji Hospital, Shanghai Jiaotong University School of Medicine, Shanghai, 200127 China; 5 Department of General Surgery, Xinhua Hospital Affiliated to Shanghai Jiao Tong University School of Medicine, 1665, Kongjiang Road, Shanghai, 200092 China

**Keywords:** Gallbladder cancer, tRNA-derived fragments, Cell cycle, Cell proliferation

## Abstract

**Background:**

tRNA-derived fragments (tRFs) are newly discovered noncoding RNAs and regulate tumor progression via diverse molecular mechanisms. However, the expression and biofunction of tRFs in gallbladder cancer (GBC) have not been reported yet.

**Methods:**

The expression of tRFs in GBC was detected by tRF and tiRNA sequencing in GBC tissues and adjacent tissues. The biological function of tRFs was investigated by cell proliferation assay, clonal formation assay, cell cycle assay, and xenotransplantation model in GBC cell lines. The molecular mechanism was discovered and verified by transcriptome sequencing, fluorescence in situ hybridization (FISH), target gene site prediction, and RNA binding protein immunoprecipitation (RIP).

**Results:**

tRF-3013b was significantly downregulated in GBC compared with para-cancer tissues. Decreased expression of tRF-3013b in GBC patients was correlated with poor overall survival. Dicer regulated the production of tRF-3013b, and its expression was positively correlated with tRF-3013b in GBC tissues. Functional experiments demonstrated that tRF-3013b inhibited GBC cell proliferation and induced cell-cycle arrest. Mechanically, tRF-3013b exerted RNA silencing effect on TPRG1L by binding to AGO3, and then inhibited NF-κB. TPRG1L overexpression could rescue the effects of tRF-3013b on GBC cell proliferation.

**Conclusions:**

This study indicated that Dicer-induced tRF-3013b inhibited GBC proliferation by targeting TPRG1L and repressed NF-κB, pointing to tRF-3013b as a novel potential therapeutic target of GBC.

**Supplementary Information:**

The online version contains supplementary material available at 10.1186/s11658-022-00398-6.

## Introduction

Gallbladder cancer (GBC) is a rare but highly malignant carcinoma that originates in the biliary tract [1, 2]. With the transformation of diet and the aging of the population, the incidence of GBC is increasing annually [3]. Due to inconspicuous early clinical symptoms, few patients were diagnosed at the early stage and the majority of patients with advanced GBC lost the opportunity for radical surgery [4, 5]. Palliative chemotherapy had limited efficacy, and most patients swiftly developed chemoresistance [6, 7]. Consequently, GBC patients had extremely poor prognosis with a 5-year survival rate of less than 15% [8]. At present, we still lack adequate understanding of its pathogenesis, and there is no specific biomarker or therapeutic approach.

Over the past few decades, various noncoding RNAs have been proven to regulate tumor progression via a variety of mechanisms, including long noncoding RNAs, microRNAs, and circular RNAs [9–11]. With the evolution of microarray technologies and high-throughput sequencing, more types of noncoding RNAs are being discovered [12]. tRNA-derived fragments (tRFs), which map to the tRNA transcripts, are newly detected noncoding RNAs that are widely present in plants, viruses, bacteria, and mammals [13–15]. Although the biogenic process of tRFs remains unclear, growing evidence suggests that they regulate translation, stress response, RNA stability, and ribosome biogenesis [16, 17]. For example, a class of tRFs suppressed breast cancer progression via YBX1 replacement [18]. In liver cancer, 3′-tRF produced by tRNA-Leu-CAG enhanced the translation of ribosomal protein RPS28 and regulated ribosome biogenesis [19]. However, no study has yet focused on the association between tRFs and GBC.

In this study, tRF and tiRNA sequencing was performed in GBC and para-cancer tissues. Subsequently, we discovered that Dicer-induced tRF-3013b suppressed GBC proliferation and induced G1/S phase arrest via downregulating expression of TPRG1L and repressing NF-κB. Our research may provide new perspectives into the mechanism of tRFs in GBC tumorigenesis.

## Methods

### Patients and clinical specimens

This study was approved by the ethics committee of Renji Hospital. Human GBC samples and para-cancer tissues were obtained from patients who underwent radical cholecystectomy without prior radiotherapy or chemotherapy between 2017 and 2019 at the Department of Biliary-Pancreatic Surgery, Renji Hospital. Patients with pathological diagnosis of gallbladder adenocarcinoma were included in this study. Each patient was pathologically staged according to the 8th edition of the AJCC TNM classification of malignant tumors. All tissues were frozen in liquid nitrogen. All patients were informed about the purpose and content of the study and signed informed consent forms.

### RNA extraction and quantitative real-time PCR

TRIzol reagent (Invitrogen, Carlsbad, CA, USA) was used to extracted total RNA. PrimeScript RT Master Mix Kit (Takara, Japan) was used for complementary DNA (cDNA) synthesis. SYBR Green (Takara, Japan) was used to perform quantitative real-time PCR (qRT-PCR). The amplified transcript level of target gene was normalized to glyceraldehyde 3-phosphate dehydrogenase (GAPDH). For tRF quantification, cDNA was synthesized using Mir-X miRNA First-Strand Synthesis (Takara, Japan), and amplification of U6 was used for normalization. All primers used for qRT-PCR are summarized in Additional file 1: Table S1.

### Cell culture

NOZ (cat. no. JCRB1033) was purchased from the Health Science Research Bank (Osaka, Japan). GBC-SD (cat. no. KCB 2011087YJ) was purchased from the Chinese Academy of Sciences (Shanghai, China). NOZ and GBC-SD cells were cultured in Dulbecco’s modified Eagle’s medium (DMEM; Gibco, USA) with 10% fetal bovine serum (Gibco, USA) and 100 µg/mL streptomycin and 100 U/mL penicillin (Gibco, USA) at 37 °C with 5% CO_2_.

### RNA oligonucleotides, plasmids, and cell transfection

RNA oligonucleotides and siRNAs were purchased from GenePharma (Shanghai, China). All RNA oligos used in this study are summarized in Additional file 2: Table S2. tRF-3013b, and negative control (NC) sequences were inserted into pLKO.1-TRC cloning vector for stable cell line construction. pLX304-Blast-V5-TPRG1L and vector were purchased from Shanghai Jiao Tong University School of Medicine. Also, 1 µg of plasmid or 50 nmol/L of RNA oligonucleotide was transfected using Lipofectamine 2000 (Invitrogen, USA).

### Cell proliferation

Cell Counting Kit-8 (Yeasen, Shanghai, China) was used to evaluate cell proliferation at 0, 1, 2, 3, 4, and 5 days. For clone formation, 500 cells were plated into a six-well culture plate and cultured for 14 days.

### Cell cycle analysis

Treated cells were collected and fixated in ice-cold 70% ethanol overnight. Cell Cycle Analysis Kit (Yeasen, Shanghai, China) was used to stain for subsequent flow cytometry. All data were analyzed using Modfit LT 5 (Verity Software).

### Western blot

Proteins were analyzed by standard Western blotting protocol. The following antibodies were used in this study: β-tubulin (A12289, Abclonal), c-myc (#18583, CST), p-CDK2 (Thr160, #2561S, CST), CDK2 (A18000, Abclonal), puromycin (EQ0001, kerafast), TPRG1L (A15949, Abclonal), p65 (#8242, CST), and p50 (A6667, Abclonal).

### Xenograft model

The animal study was approved by the Institutional Animal Care and Use Committee of Xinhua Hospital. Male BALB/c nude mice (4–6 weeks old) were purchased from the Shanghai Laboratory Animal Center of the Chinese Academy of Sciences (Shanghai, China). A total of 1 × 10^6^ NOZ cells (Lv-NC, Lv-tRF-3013b) were inoculated subcutaneously into the inguinal region. Tumor volumes were measured every 5 days as 1/2 × length × width^2^. Mice were euthanized, and the weights of tumors were measured approximately 3 weeks later.

### Fluorescence in situ hybridization (FISH)

The 3′ CY3-labeled antisense locked nucleic acid-modified probe was synthesized by GenePharma (Shanghai, China). FISH was performed according to the manufacturer’s instruction (GenePharma, Shanghai, China).

### Translation analysis

After 48 h of transfection, treating cells with fresh DMEM containing 10 µg/mL puromycin for 5 min at 37 °C before harvesting. The incorporation of puromycin and nascent peptides was detected by anti-puromycin (kerafast, USA).

### Dual-luciferase reporter assay

The TPRG1L-3′UTR-WT/MUT plasmids were cloned into GV272. 293T cells cotransfected with NC/mimic and GV272-TPRG1L-3′UTR-WT/MUT plasmids. After 48 h, the luciferase activity was detected according to the manufacturer’s instructions (Yeasen, Shanghai, China).

### RNA immunoprecipitation (RIP) assay

RIP was performed using the Magna RIP RNA-binding Protein Immunoprecipitation Kit (Millipore, Bedford, MA). Ago2 and Ago3 antibody were purchased from Sigma-Aldrich. Total RNA (input) and IgG control were simultaneously assayed.

### Immunohistochemistry (IHC)

Xenograft tumors were fixated in 4% polyoxymethylene for 3 days, embedded in paraffin, and then cut to 5 mm thickness. After dewaxing, rehydration, and antigen retrieval, slides were incubated with anti-Ki-67 (A2094, Abclonal) and anti-TPRG1L (A15949, Abclonal).

### Statistical analysis

The variation between two groups was compared using Student’s *t*-test. The variation among three or more groups was compared using one-way analysis of variance (ANOVA) test. Kaplan–Meier curves and log-rank tests were used to evaluate the survival curves among groups. All data were analyzed using SPSS v21.0 and GraphPad Prism 8. *P* < 0.05 was considered significant.

## Results

### tRF-3013b was significantly downregulated in GBC and correlated with prognosis

**Fig. 1:**
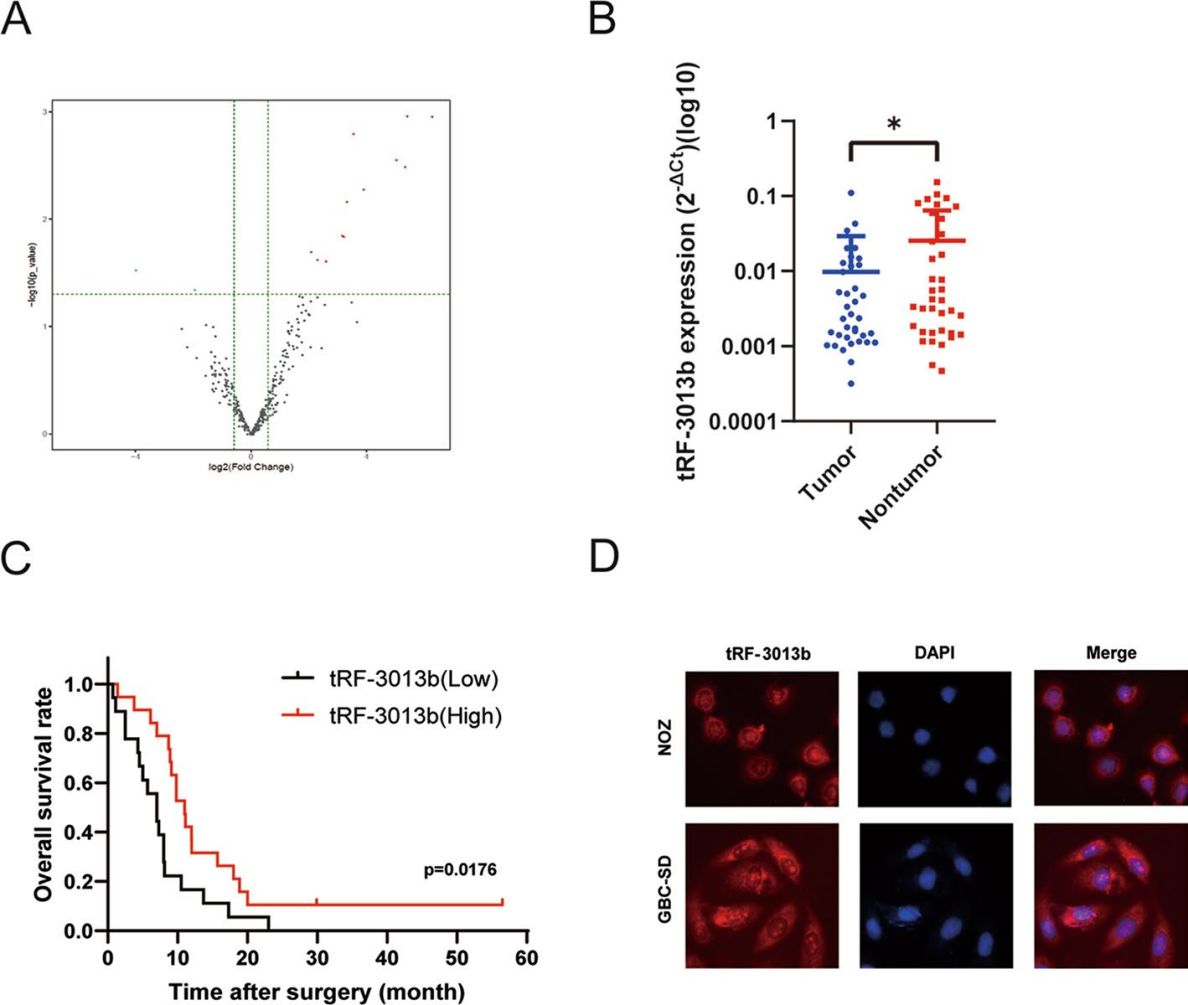
tRF-3013b was significantly downregulated in GBC and correlated with prognosis. **A** Volcano plot of tRF and tiRNA. Green/red circles indicate differentially expressed tRFs and tiRNAs with fold change no less than 1.5 and *P* ≤ 0.05 (green: downregulated; red: upregulated). **B** tRF-3013b expression level in 37 pairs of GBC and para-cancer tissues. **C** Overall survival of GBC patients with high or low tRF-3013b expression. **D** Location of tRF-3013b in NOZ and GBC-SD cells detected by FISH. *P < 0.05

To explore tRFs expression in GBC, tRF and tiRNA sequencing was performed on four pairs of GBC and para-cancer tissues (data deposited in NODE with accession number OEP003077, https://www.biosino.org/node/index). tRFs with lengths of 20–23 nt and 30–33 nt were relatively abundant in GBC (Additional file 3: Fig. S1A). The pie chart showed the proportional distribution of each tRF subtype, where the average counts per million of total aligned reads (CPM) of the group or the CPM of the sample was not less than 20 (Additional file 3: Fig. S1B). Among 321 detected tRFs, 12 were significantly upregulated and 2 were significantly downregulated in GBC tissues compared with para-cancer tissues (Fig. 1A; Table 1). We focused on downregulated 3′-tRF-His-GTG-012 (22 nt, derived from tRNA-His-GTG), which transports histidine, having anticodon of GTG; tRFdb ID: tRF-3013b (http://genome.bioch.virginia.edu/trfdb/index.php) in subsequent investigation owing to its relatively higher CPM value and the lack of studies reporting its biofunction.

**Table 1:** Differentially expressed tRFs in GBC

tRF_ID	tRFdb_ID	Sequence of tRF	Alignment information	log_2_FC
Upregulated				
tRF-Lys-CTT-002	5005a	GCCCGGCTAGCTCAG	tRNA-Lys-CTT	6.265838
tRF-Lys-TTT-003	–	GCCCGGATAGCTCAG	tRNA-Lys-TTT	5.407817
tRF-Ile-AAT-002	5010a	GGCCGGTTAGCTCAG	tRNA-Ile-AAT	5.333084
tRF-Tyr-GTA-015	5001a	CCTTCGATAGCTCAG	tRNA-Tyr-GTA	5.027101
tRF-Val-TAC-010	–	GGTTCCATAGTGTAG	tRNA-Val-TAC	3.900503
tRF-Tyr-GTA-002	–	CCTTCGATAGCTCAGC	tRNA-Tyr-GTA	3.542264
tRF-Val-CAC-004	5009a	GCTTCTGTAGTGTAG	tRNA-Val-CAC	3.314453
tRF-Ser-AGA-003	–	GTAGTCGTGGCCGAG	tRNA-Ser-AGA	3.207555
tRF-SeC-TCA-001	–	GCCCGGATGATCCTC	tRNA-SeC-TCA	3.155524
tRF-Glu-TTC-007	–	TCCCACATGGTCTAGC	tRNA-Glu-TTC	2.597704
tRF-Val-AAC-005	–	GTTTCCGTAGTGTAG	tRNA-Val-AAC	2.300519
tRF-Gly-CCC-007	5004b	GCATTGGTGGTTCAGTGGTAGA	tRNA-Gly-CCC	2.078226
Downregulated				
tRF-Val-TAC-023	–	AACTTACACTTAGG	tRNA-Val-TAC	− 3.98713
tRF-His-GTG-012	3013b	TCGAATCCGAGTCACGGCACCA	tRNA-His-GTG	− 1.94414

To verify the expression of tRF-3013b in GBC, its expression level in 37 pairs of GBC tissues and para-cancer tissues was detected using qPCR. Consistent with tRF and tiRNA sequencing results, tRF-3013b was significantly downregulated in GBC tissues relative to para-cancer tissues (Fig. 1B). Moreover, patients with lower expression of tRF-3013b had worse overall survival (Fig. 1C). Next, the correlation between tRF-3013b and clinicopathological status was evaluated (Table 2). Possibly owing to the limited number of patients or other confounding factors, the percentage of patients with T3–4 stage or lymph node metastases was higher in the lower tRF-3013b expression group, although this did not reach significance.

**Table 2:** Correlation between clinicopathological status and expression of tRF-3013b

Variable	Category	Relative tRF-3013b expression		*χ*^2^	*P*
		Low (*n* = 18)	High (*n* = 19)		
Age (years)	< 60	6	8	0.302	0.737
	≥ 60	12	11		
Gender	Male	6	5	0.218	0.728
	Female	12	14		
Histological differentiation	Poor	8	13	0.650	0.508
	Moderate or well	10	6		
T stage	Tis-T_2_	4	9	2.565	0.170
	T_3_–T_4_	14	10		
Lymph node metastasis	Present	13	8	3.416	0.099
	Absent	5	11		

### tRF-3013b originates from tRNA and is regulated by Dicer

**Fig. 2:**
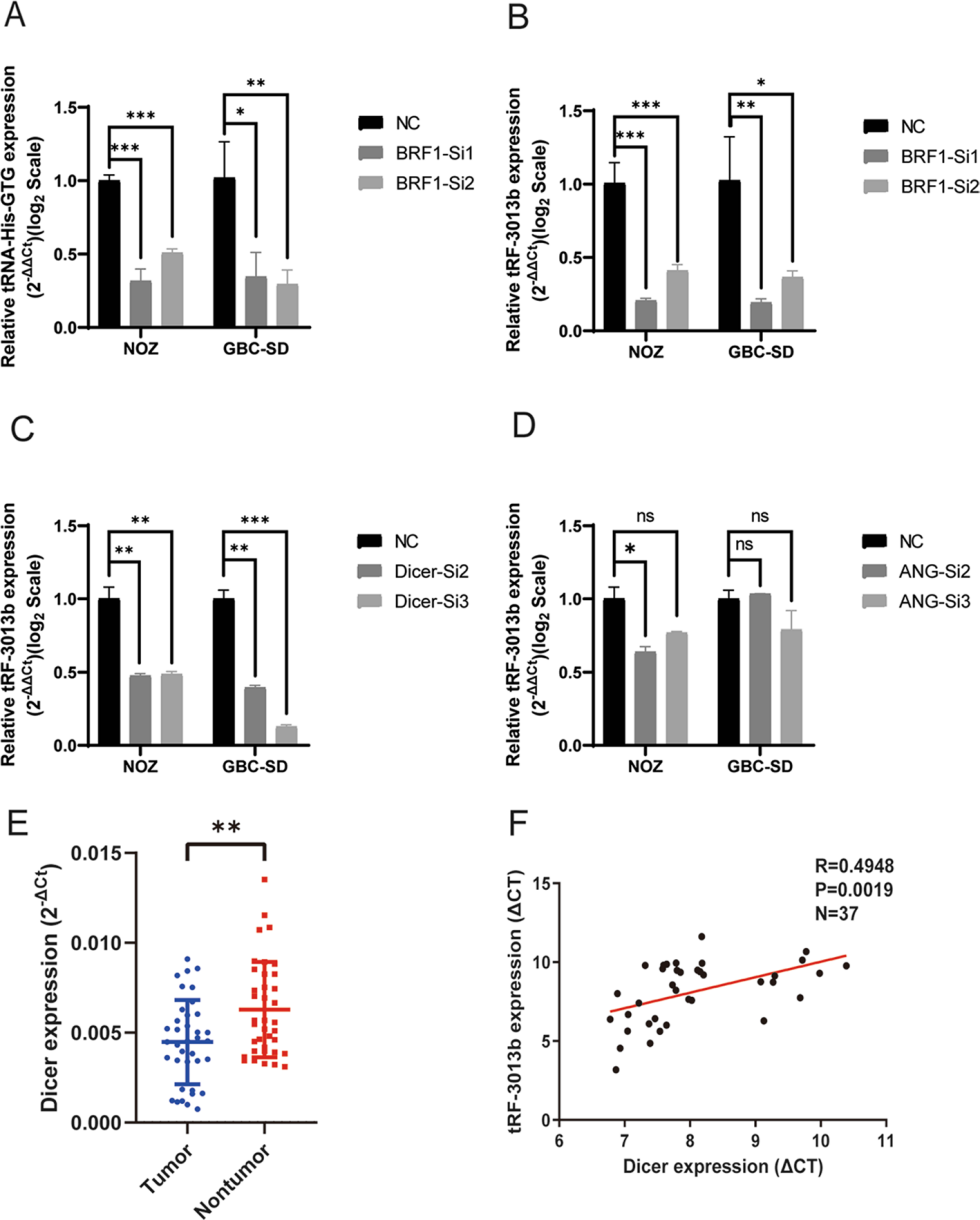
Dicer induced production of tRF-3013b in GBC cells. **A** Expression of tRNA-His-GTG in GBC cells with BRF1 knockdown. **B** Expression of tRF-3013b in GBC cells with BRF1 knockdown. **C** Expression of tRF-3013b in GBC cells with Dicer knockdown. **D** Expression of tRF-3013b in GBC cells with ANG knockdown. **E** Dicer expression level in 37 pairs of GBC and para-cancer tissues. **F** Linear regression analysis and paired *t*-test for determining the correlation between the expression levels of tRF-3013b and Dicer. ns *P* > 0.05, *P < 0.05, **P < 0.01, ***P < 0.001

To verify the expression of tRF-3013b in GBC cells, FISH was performed, and it was observed that tRF-3013b expressed in both cytoplasm and nucleus (Fig. 1D). Subsequently, we further explored the generation of tRF-3013b. BRF1, a subunit of the RNA polymerase III transcription factor complex, plays a critical role in the initiation of tRNA transcription [20]. Knockdown of BRF1 results in an inability for tRNA transcription. To further prove that tRF-3013b originated from tRNA, BRF1 was knocked down in NOZ and GBC-SD cells, respectively (Additional file 4: Fig. S2A, B). As a result, the relative expression of tRNA-His-GTG and tRF-3013b were significantly decreased after BRF1 knockdown (Fig. 2A, B), suggesting that tRF-3013b was derived from tRNA.

Previous studies have determined that RNases were involved in the process of cleaving tRNAs and generating tRFs [21]. Among them, angiogenin (ANG) could cleave the anticodon site of tRNA, and Dicer could fragment T loop and D loop of tRNA [22–25]. To investigate whether the generation of tRF-3013b was mediated by RNases, ANG and Dicer were knocked down in NOZ and GBC-SD cells, respectively (Additional file 4: Fig. S2C–F). Consequently, relative expression of tRF-3013b was significantly decreased after Dicer knockdown (Fig. 2C). Meanwhile, there was no such phenotype after ANG knockdown (Fig. 2D), suggesting that Dicer was involved in the generation of tRF-3013b but not ANG. To further determine the correlation between tRF-3013b and Dicer, we detected the expression of Dicer in previous 37 pairs of GBC tissues and para-cancer tissues using qPCR. In comparison with para-cancer tissues, Dicer was significantly downregulated in GBC tissues and its expression was positively correlated with tRF-3013b expression (Fig. 2E, F). These results indicated that Dicer was involved in the generation of tRF-3013b.

### tRF-3013b inhibits GBC cell proliferation and induces G1/S phase arrest

**Fig. 3:**
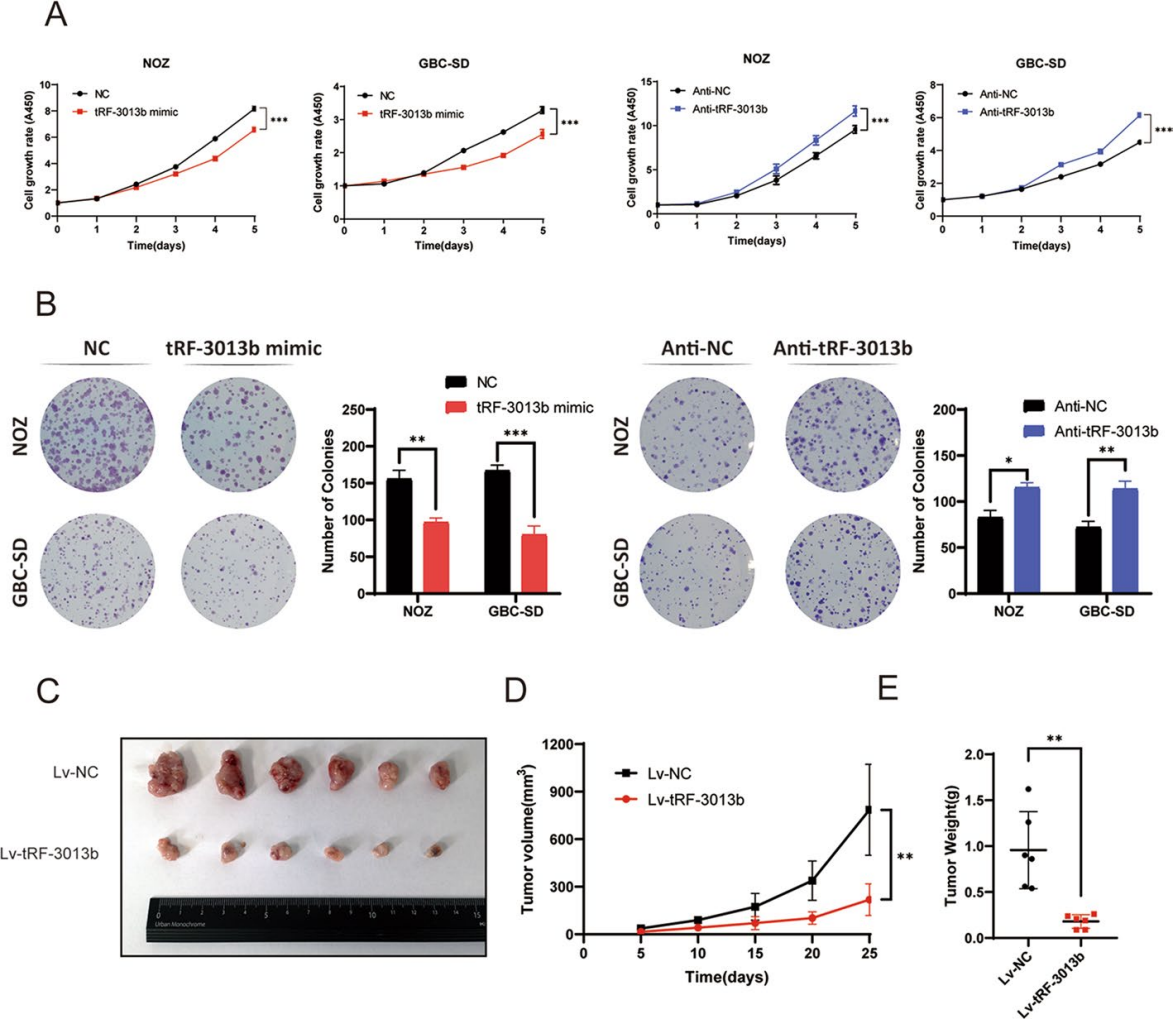
tRF-3013b inhibited GBC cell proliferation. **A** CCK8 assay and **B** colony formation were performed to evaluated proliferation capacity after overexpression and inhibition of tRF-3013b in NOZ and GBC-SD cells. **C** Xenograft size of NOZ-transfected Lv-NC or LV-tRF-3013b. **D** Tumor volume was detected to supervise tumor growth every 5 days. **E** Tumor weight was calculated and compared in subcutaneous implantation mouse models. *P < 0.05, **P < 0.01, ***P < 0.001

Next, we sought to investigate the biofunction of tRF-3013b in GBC cell proliferation and tumorigenesis. tRF-3013b mimic and its specific inhibitor anti-tRF-3013b were transfected into GBC-SD and NOZ cells, respectively (Additional file 5: Fig. S3). By CCK8 and colony-formation assays, it was observed that tRF-3013b mimic significantly inhibited GBC cell proliferation compared with NC. Simultaneously, anti-tRF-3013b significantly promoted GBC cell proliferation relative to anti-NC (Fig. 3A, B), suggestive of the inhibitory effect of tRF-3013b in GBC cells. To validate the observation in vivo, NOZ cells stably overexpressing tRF-3013b or NC were constructed and used in the xenograft model. In agreement with the in vitro data, tRF-3013b significantly inhibited tumor growth compared with NC (Fig. 3C–E). Moreover, IHC showed reduced number of Ki-67 positive cells in Lv-tRF-3013b-inoculated tumor tissues (Fig. 5F). Collectively, these results demonstrated that tRF-3013b acted as an inhibitory factor in GBC cells.

**Fig. 4:**
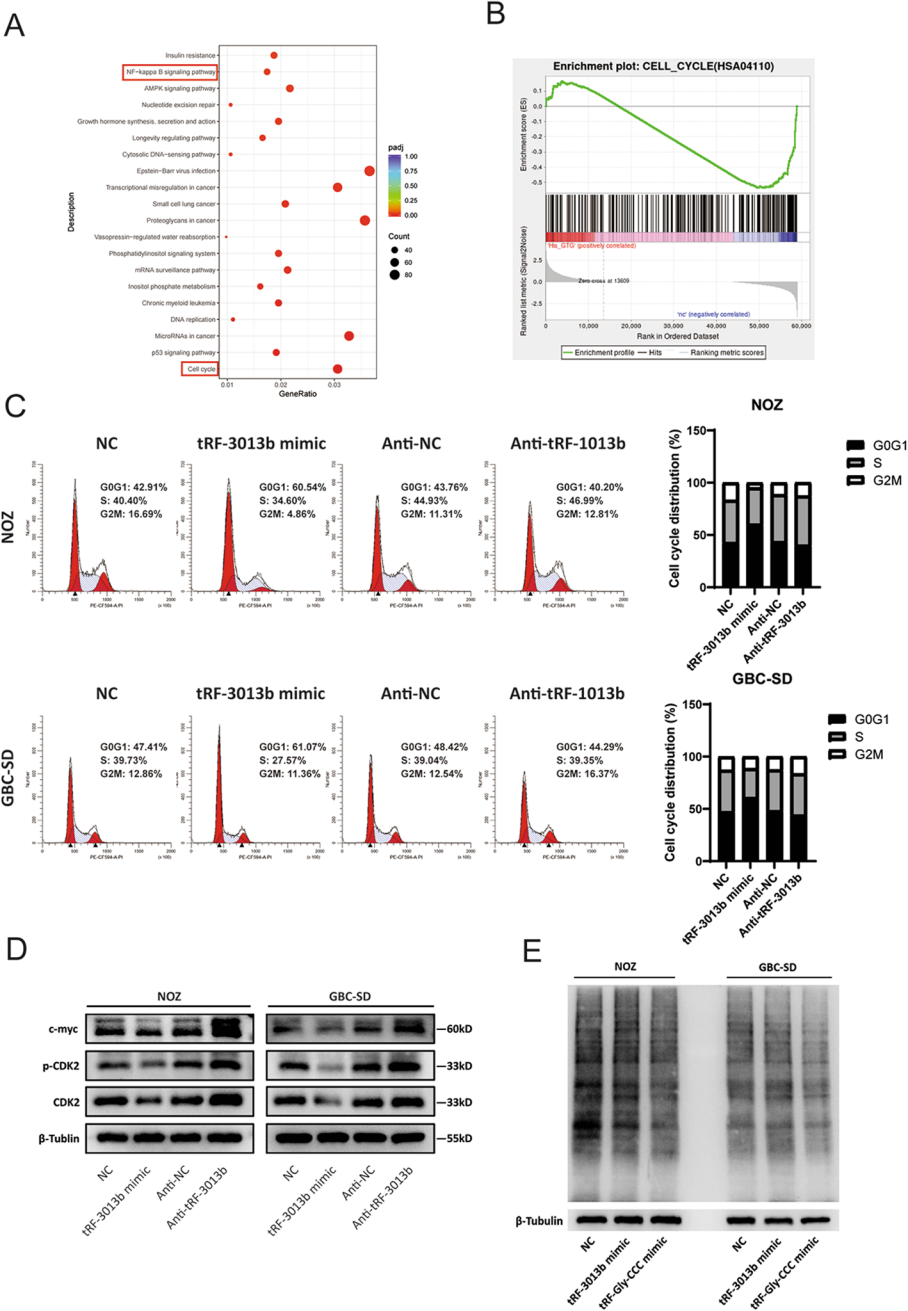
tRF-3013b induced G1/S phase arrest in GBC cells. **A** KEGG rich distribution map showing the related pathways after transfection of NOZ with NC or tRF-3013b mimic. **B** Enrichment plot of cell cycle analyzed with GSEA after transfection with NC or tRF-3013b mimic in NOZ cells. **C** Cell-cycle assay after overexpression and inhibition of tRF-3013b in NOZ and GBC-SD cells. **D** Relative protein expression of c-myc, CDK2, and p-CDK2 after overexpression and inhibition of tRF-3013b in NOZ and GBC-SD cells. **E** Incorporation of puromycin and nascent peptides was detected to evaluated translation progression after transfection with NC, tRF-3013b mimic, or tRF-Gly-CCC mimic (positive control) in NOZ and GBC-SD cells

To further determine the effect of tRF-3013b on GBC cell proliferation, we performed RNA-seq to compare and analyze the gene expression profiles of tRF-3013b mimic and NC transfectants in NOZ cells. A total of 5869 differential genes were detected (≥ twofold) between tRF-3013b mimic and NC transfectants in NOZ cells (Additional file 6: Fig. S4A, B). Kyoto Encyclopedia of Genes and Genomes (KEGG) revealed that tRF-3013b was related to cell cycle (Fig. 4A). Gene Ontology (GO) enrichment analysis revealed that tRF-3013b was involved in mitotic cell cycle phase (Additional file 6: Fig. S4C, D), and Gene Set Enrichment Analysis (GSEA) also indicated that tRF-3013b was related to CELL_CYCLE (Fig. 4B), suggesting that tRF-3013b might be a modulator in GBC cell cycle. Therefore, we speculated that tRF-3013b inhibited GBC cell proliferation by arresting cell cycle. As shown in Fig. 4C, overexpression of tRF-3013b led to increased proportion of G0G1 phase cells while inhibition of tRF-3013b resulted in a decrease in the ratio of G0G1 phase cells, indicating that tRF-3013b induced G1/S phase arrest in GBC cells. Consistent with cell-cycle distribution, c-myc and CDK2, as key genes in G1/S phase transition [26–28], were negatively regulated by tRF-3013b (Fig. 4D), suggesting that tRF-3013b might arrest cell cycle by regulating c-myc and CDK2.

### TPRG1L is a direct target gene of tRF-3013b

We next explored the molecular mechanism of tRF-3013b in inhibiting GBC cell proliferation. Given that tRFs originate from tRNAs, which play a critical role in the translation process and that some types of tRFs, such as tRF-Gly, have been found to inhibit the cellular global translation process by competitively binding to translation initiation factor eIF4G/eIF4A [24]. We detected whether tRF-3013b inhibited GBC proliferation via affecting the translation process in GBC cells. As shown in Fig. 4E, unlike tRF-Gly-CCC (positive control), tRF-3013b did not significantly regulate the global translation function in GBC cells.

**Fig. 5:**
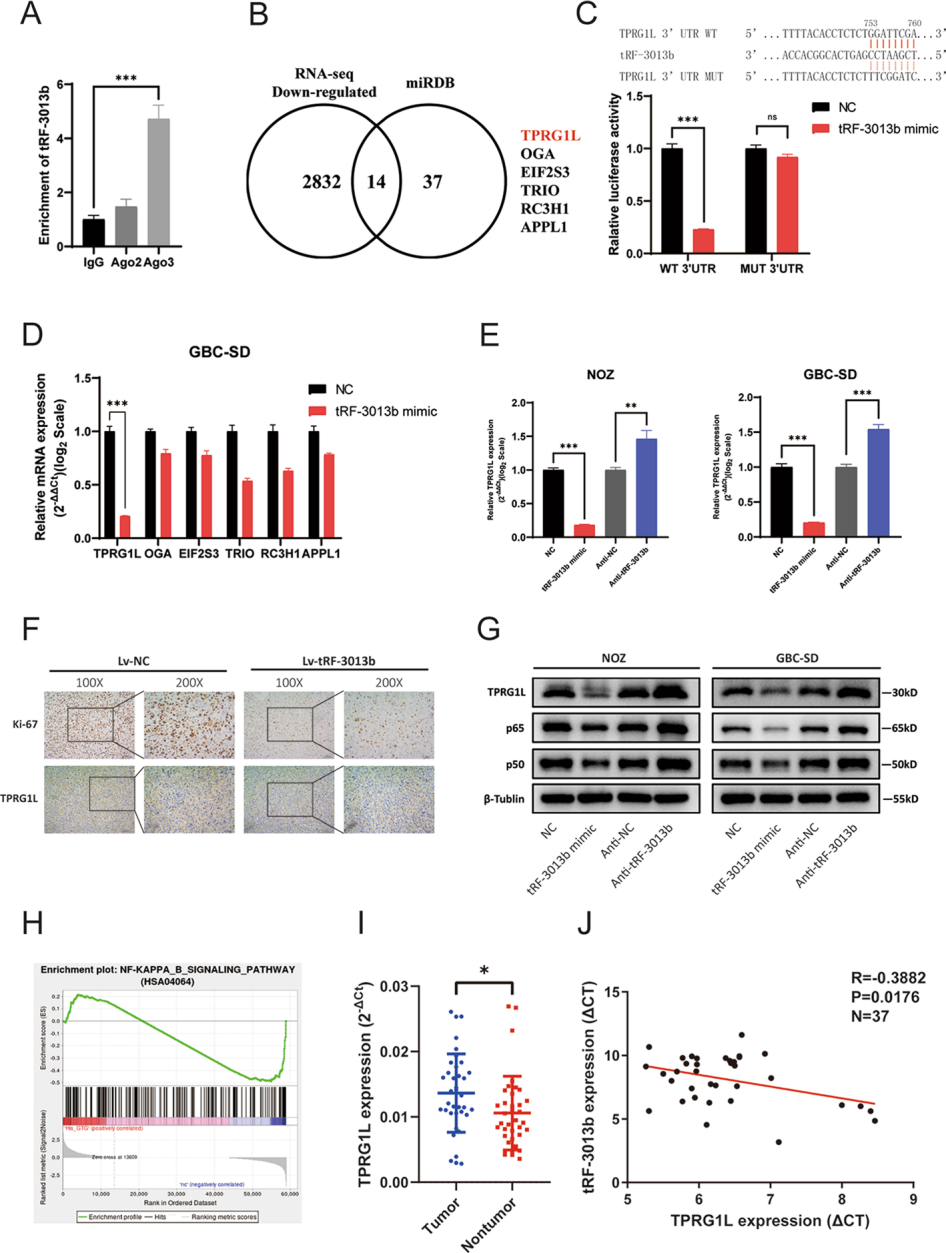
TPRG1L was a direct target of tRF-3013b. **A** The enrichment between AGO2, AGO3, and tRF-3013b was detected by RIP-qPCR. **B** Potential tRF-3013b targets intersected by RNA-seq and miRDB. **C** Sequence of the predicted tRF-3013b binding site within the 3′-UTR of TPRG1L. Relative luciferase activity was detected after cotransfection of NC or tRF-3013b mimic and reporter plasmids into 293T cells. **D** Relative mRNA expression of potential target genes after transfection with NC or tRF-3013b mimic in GBC cells. **E** Relative mRNA expression of TPRG1L after overexpression and inhibition of tRF-3013b NOZ and GBC-SD cells. **F** IHC showing Ki-67 and TPRG1L protein expression in xenograft transfected Lv-NC or LV-tRF-3013b. **G** Relative protein expression of TPRG1L, p65, and p50 after overexpression and inhibition of tRF-3013b in NOZ and GBC-SD cells. **H** Enrichment plot of NF-κB signaling pathway analyzed with GSEA after transfection with NC or tRF-3013b mimic in NOZ cells. **I** TPRG1L expression level in 37 pairs of GBC and para-cancer tissues. **J** Linear regression analysis and paired *t*-test for determining the correlation between the expression levels of tRF-3013b and TPRG1L. ns *P* > 0.05, *P < 0.05, **P < 0.01, ***P < 0.001

Growing evidence indicates that the combination of tRFs with argonaute (AGO) mediates the degradation of target mRNA by RNA-degrading enzymes [16]. Previous studies have shown that tRFs prefer to bind to AGO3 and participate in gene expression regulation [29, 30]. By RIP-qPCR analysis, we observed that AGO3 significantly enriched tRF-3013b (Fig. 5A), indicating that tRF-3013b might regulate oncogene expression by binding to AGO3. To explore the potential target genes of tRF-3013b, downregulated genes detected in RNA-seq and miRDB target-prediction programme (http://mirdb.org/mirdb/index.html) were intersected. Among 14 intersecting genes, we selected 6 that might be related to tumor progression for further verification (Fig. 5B). Base on qPCR verification in GBC-SD cells, *TPRG1L* was focused as a target gene of tRF-3013b in subsequent experiments because it was significantly downregulated after tRF-3013b overexpression (Fig. 5D). We then predicted potential binding sites of tRF-3013b in the 3′-UTR of TPRG1L mRNA using miRDB (Fig. 5C). Hence, the TPRG1L 3′UTR wild-type (WT) sequence and mutant tRF-3013b binding sites sequence were cloned into the GV272 vector for dual luciferase reporter assay. The results indicated that tRF-3013b repressed TPRG1L-3′UTR-WT luciferase reporter expression but did not affect TPRG1L-3′-UTR-MUT luciferase reporter expression (Fig. 5C). qPCR, IHC, and Western blot also revealed that tRF-3013b inhibited endogenous TPRG1L mRNA and protein expression (Fig. 5E–G). Moreover, TPRG1L was significantly upregulated in GBC tissues in comparison with para-cancer tissues and negatively correlated with tRF-3013b (Fig. 5I, J). In accordance with our findings, GBC patient tumors with low tRF-3013b expression showed strong TPRG1L staining, whereas tumors with high tRF-3013b expression showed weak TPRG1L IHC staining (Additional file 7: Fig. S5D). In general, these results indicated that tRF-3013b directly targeted TPRG1L 3′UTR region and negatively regulated its expression.

TPRG1L is a cytoplasmic protein widely expressed in various tissues, but few studies have focused on its biofunction. According to reports, TPRG1L was an activator of NF-κB pathway [31, 32], which could participate in cell-cycle regulation [33]. In this study, KEGG and GSEA analyses also revealed that tRF-3013b is related to NF-κB signaling pathway (Figs. 4A, 5H). Hence, we detected whether NF-κB was regulated by tRF-3013b. The results indicated that expression of p65 and p50 was negatively regulated by tRF-3013b (Fig. 5G), suggesting that tRF-3013b might inhibit expression of TPRG1L and subsequently repress NF-κB.

### TPRG1L overexpression rescues the phenotypes of tRF-3013b on GBC cell proliferation

**Fig. 6:**
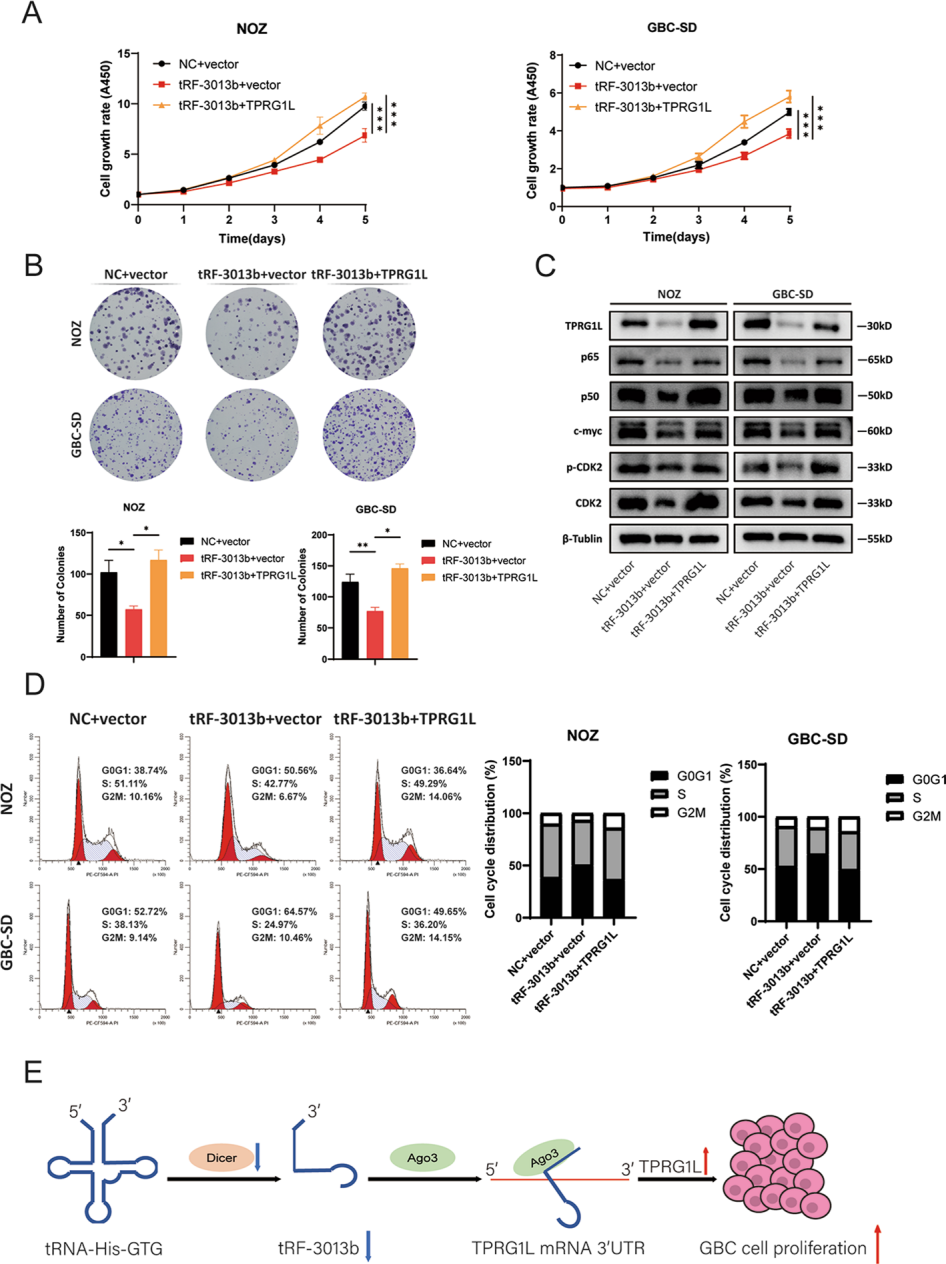
Overexpression of TPRG1L rescued the inhibitory effects of tRF-3013b. **A** CCK8 assay and **B** colony formation were performed to evaluated cell proliferation capacity after cotransfection of NOZ and GBC-SD with NC or tRF-3013b mimic and vector or TPRG1L. **C** Relative protein expression of TPRG1L, p65, p50, c-myc, CDK2, and p-CDK2 after cotransfection of NOZ and GBC-SD with NC or tRF-3013b mimic and vector or TPRG1L. **D** Cell-cycle assay was evaluated after cotransfection of NOZ and GBC-SD with NC or tRF-3013b mimic and vector or TPRG1L. **E** Schematic depiction of the function and potential mechanism of tRF-3013b in GBC. *P < 0.05, **P < 0.01, ***P < 0.001

To further evaluate whether the inhibition of GBC cell proliferation by tRF-3013b was indeed mediated by TPRG1L, we examined the function of TPRG1L in GBC cells and found that overexpression of TPRG1L promoted GBC cell proliferation (Additional file 7: Fig. S5A, B). Western blot analyses confirmed that expression of p65, p50, c-myc, CDK2, and p-CDK2 was enhanced by overexpression of TPRG1L (Additional file 7: Fig. S5C). Next, NOZ and GBC-SD were cotransfected with tRF-3013b mimic or NC together with pLX304-vector or pLX304-TPRG1L. It was observed that overexpression of TPRG1L reversed the proliferation and cell-cycle phenotype caused by overexpression of tRF-3013b (Fig. 6A, B, D). Furthermore, overexpression of TPRG1L rescued the downregulation of p65, p50, c-myc, and CDK2 caused by overexpression of tRF-3013b (Fig. 6C), suggesting that tRF-3013b targeted TPRG1L degradation and subsequently inhibited GBC cell proliferation by repressing NF-κB. Collectively, all of these observations suggest that tRF-3013b was regulated by Dicer and inhibited GBC cell proliferation via downregulating expression of TPRG1L (Fig. 6E).

## Discussion

Over the past few decades, noncoding RNAs have been proven to participate in the progression of cancer via a variety of biological mechanisms. tRFs, derived from tRNAs, are a new class of noncoding RNAs discovered recently [34]. It has been noticed that tRFs are involved in various diseases, including inflammation, virus infection, metabolic disorder, and cancer [13]. In the current study, we screened tRFs expression between GBC and para-cancer tissues for the first time and discovered that tRF-3013b was associated with GBC.

Mature tRNA is 73–90 nt in length and forms a tightly condensed cloverleaf-shaped secondary structure [15]. The cloverleaf-shaped tRNA folded into a tight L-shape in three dimensions [29]. The anticodon, T loop, and D loop were relatively exposed sites and could be fragmented by RNases [21]. ANG cleaved the anticodon to generate ~ 30 nt fragments, while Dicer was related to the cleavage of T loop and D loop to produce ~ 18–22 nt fragments, whereas cleavage at other sites is less abundant [21, 35]. In the current study, the detected tRFs in GBC were also clustered in ~ 22 nt and ~ 31 nt (Fig. 1A), consistent with previous reports. tRF-3013b is 22 nt in length, located from T-loop to 3′ tRNA-His-GTG. Theoretically, its production was mediated by Dicer. In this regard, we conducted a brief verification and found that its expression level was downregulated by Dicer knockdown but not ANG (Fig. 2C, D), suggesting that the generation of tRF-3013b was regulated by Dicer-mediated T loop cleavage. This phenomenon is consistent with other previous studies [22, 23].

Dicer is a member of the RNase III family as a key factor in the generation of small regulatory RNAs [36, 37]. Deregulation of Dicer has been observed in a variety of tumors, including ovarian cancer [38], breast cancer [39], and lung cancer [40]. Compared with non-dysplastic gallbladder epithelia, Dicer expression was significantly lower in GBC and loss of Dicer was an independent poor prognostic predictor in GBC patients [41]. Here, we also observed that Dicer showed lower expression in GBC relative to para-cancer tissues. Additionally, expression of Dicer was positively correlated with tRF-3013b in GBC tissues.

Accumulating evidence indicates that tRFs act as suppressors or oncogenes in tumor genesis and progression. For example, tRF-1280 suppressed metastasis and stem-cell-like cells in colorectal cancer [42]. tRF-03357 promoted ovarian cancer proliferation and metastasis [43]. In this study, we found that tRF-3013b repressed GBC cell proliferation and tumor growth. From the RNA-seq perspective, the antiproliferative effect of tRF-3013b seemed to be associated with cell cycle. Flow cytometry demonstrated that tRF-3013b could arrest GBC cells in G1/S phase. In the process of mitosis, c-myc plays a critical role in cell proliferation and cell cycle progression [44]. In addition to regulating glycolysis, protein synthesis, nucleotide metabolism, and DNA replication in preparation for cell division, c-myc also participates in G1/S phase transition by activating cyclins [45, 46]. CDK2 is a key cyclin-dependent kinase that drives G1/S phase transition [47, 48]. In the current study, we observed that tRF-3013b negatively regulated expression of c-myc and CDK2, suggesting that tRF-3013b might arrest cell cycle by c-myc and CDK2.

Mechanically, tRFs could displace translation eukaryotic initiation factor to regulate global translation or bind to AGO and function as RNA silencing [16]. We detected the effect of tRF-3013b on global translation, but a negative representation appeared. We then speculated that tRF-3013b inhibited GBC cell proliferation via binding AGO and inhibiting oncogene expression. Among AGO family proteins, tRFs preferentially bind to AGO3 [13, 49]. By RIP-qPCR, we demonstrated that tRF-3013b could combine with AGO3 in GBC cells. Furthermore, dual luciferase reporter assay indicated that tRF-3013b could bind with the 3′-UTR region of TPRG1L mRNA and then exert RNA silencing. qRT-PCR, IHC, and Western blot confirmed that tRF-3013b negatively regulated expression of TPRG1L. Moreover, TPRG1L was upregulated in GBC tissues and negatively correlated with tRF-3013b. Collectively, TPRG1L was a direct target gene of tRF-3013b.

TPRG1L is a cytoplasmic protein widely expressed in various tissues, but its function is not well understand [50]. In the central nervous system, TPRG1L has been found to regulate exocytosis of synaptic vesicles [51, 52]. In diabetes mellitus, TPRG1L was upregulated compared with in normal people and activated the NF-κB/IL-6 axis [31]. Analogously, it has been found that TPRG1L upregulated IL-6 expression via NF-κB pathway in human cytomegalovirus infection [32]. Therefore, we were concerned whether tRF-3013b would affect NF-κB, which acts as a mediator of cell-cycle progression in tumor cells [33, 53]. In addition, NF-κB has been reported to act as a transcriptional factor of c-myc [54, 55] and CDK2 [56]. NF-κB also participates in the cell cycle by regulating cyclins such as cyclin D1 [57, 58]. Here, we found that tRF-3013b targeted TPRG1L and subsequently downregulated expression of NF-κB, c-myc, and CDK2, suggesting that tRF-3013b targeted TPRG1L and inhibited NF-κB, which led to downregulation of cell-cycle regulatory proteins such as c-myc and CDK2, resulting in cell-cycle arrest and cell proliferation inhibition in GBC.

However, there are some issues to be resolved in this study. Few clinic samples were used for validation, and the correlation between tRF-3013b and clinicopathological information did not reach significance. This study verified that Dicer regulated tRF-3013b production but did not determine whether there was a sufficient and necessary relationship between Dicer and tRF-3013b production. Besides, the regulatory mechanism of TPRG1L on NF-κB and the cell cycle also needs further verification.

## Conclusions

Our results demonstrated that tRF-3013b was significantly downregulated in GBC. Dicer-induced tRF-3013b inhibited GBC proliferation via directly targeting TPRG1L and repressing NF-κB. The current study provides new perspectives on the molecular mechanism of tRFs and TPRG1L in GBC oncogenesis.

## Supplementary Information

**Additional file 1: Table S1.** Primers for qRT-PCR in the study.

**Additional file 2: Table S2.** RNA oligos used in the study.

**Additional file 3: Fig S1.** tRFs expression in GBC tissues. (A) Bar chart of the sequence read length distribution of tRF and tiRNA. (B) Pie chart of the distribution of subtype tRF and tiRNA.

**Additional file 4: Fig S2.** Verification of BRF1, Dicer, and ANG knockdown. (A, B) Relative mRNA expression of BRF1 in NOZ and GBC-SD cells with BRF1 knockdown. (C, D) Relative mRNA expression of Dicer in NOZ and GBC-SD cells with Dicer knockdown. (E, F) Relative mRNA expression of ANG in NOZ and GBC-SD cells with ANG knockdown. ns *P* > 0.05, **P < 0.01, ***P < 0.001.

**Additional file 5: Fig S3.** Verification of tRF-3013b overexpression and inhibition. (A, B) Relative tRF-3013b expression with NC or tRF-3013b mimic transfection in NOZ and GBC-SD cells. (C, D) Relative tRF-3013b expression with Anti-NC or Anti-tRF-3013b transfection in NOZ and GBC-SD cells. **P < 0.01, ***P < 0.001.

**Additional file 6: Fig S4.** tRF-3013b was related to the cell cycle progression. (A) Heatmap showing the differential genes between NOZ cells transfected with NC or tRF-3013b mimic. (B) Volcano plot showing the differential genes between NOZ cells transfected with NC or tRF-3013b mimic. (C) GO enrichment distribution map showing the related pathways after transfection with NC or tRF-3013b mimic in NOZ cells. (D) Significance of related pathways by GO enrichment analysis (BP, biological process; CC, cellular component; MF, molecular function).

**Additional file 7: Fig S5.** TPRG1L promoted GBC cell proliferation. (A) CCK8 assay and (B) colony formation were performed to evaluated proliferation capacity after transfection with vector or TPRG1L. (C) Relative protein expression of TPRG1L, p65, p50, c-myc, CDK2, and p-CDK2 after transfection with vector or TPRG1L. (D) Representative IHC micrographs showing TPRG1L protein expression in GBC tissues with high or low miR-143-3p expression. *P < 0.05, **P < 0.01, ***P < 0.001.

## Data Availability

The data can be obtained by email (laoniulyb@shsmu.edu.cn) under reasonable request.

## Abbreviations

tRFs: tRNA-derived fragments
GBC: Gallbladder cancer
qRT-PCR: Quantitative real-time polymerase chain reaction
GAPDH: Glyceraldehyde 3-phosphate dehydrogenase
cDNA: Complementary DNA
NC: Negative control
DMEM: Dulbecco’s modified Eagle’s medium
FISH: Fluorescence in situ hybridization
RIP: RNA immunoprecipitation
CPM: Average counts per million of total aligned reads
ANG: Angiogenin
IHC: Immunohistochemistry
KEGG: Kyoto Encyclopedia of Genes and Genomes
GO: Gene Ontology
GSEA: Gene Set Enrichment Analysis
AGO: Argonaute
WT: Wild type
