# Supplementary material for: tRF-3013b inhibits gallbladder cancer proliferation by targeting TPRG1L
Source: Cell Mol Biol Lett. 2022 Nov 18;27:99. doi: 10.1186/s11658-022-00398-6 (PMC9673407; doi:10.1186/s11658-022-00398-6)
Supplement: Supplementary file 1 — Additional file 1: Table S1. Primers for qRT-PCR in the study. [file 11658_2022_398_MOESM1_ESM.docx]

**Table S1. Primers for qRT-PCR in the study**

| **Primer name** | **sequence（5’-3’）** |
| --- | --- |
| BRF1-F | ATCTACAAGGAACACAAGCCCA |
| BRF1-R | CGGAGCACGCTATAATTGATCT |
| ANG-F | CAAGGCCATCTGTGAAAACAAG |
| ANG-R | CAGGGGGAACCTCCATGTAG |
| Dicer-F | TGCTATGTCGCCTTGAATGTT |
| Dicer-R | AATTTCTCGATAGGGGTGGTCTA |
| GAPDH-F | AGAAGGCTGGGGCTCATTTG |
| GAPDH-R | AGGGGCCATCCACAGTCTTC |
| tRNA-His-GTG-F | GCCGTGATCGTATAGTGGTTAG |
| tRNA-His-GTG-R | TGGTGCCGTGACTCGGATTC |
| tRF-3013b-F | GGTCGAATCCGAGTCACG |
| U6-F | CTCGCTTCGGCAGCACA |
| U6-R | AACGCTTCACGAATTTGCGT |
| TPRG1L-F | CAACGCAGTAGACACCATTTCC |
| TPRG1L-R | CCCAAAACCTTCTCGCTTGTT |
| OGA-F | CATAGGATGTTTTGGCGAGAGAT |
| OGA-R | GGTGAGATCGCATAGATGAACTC |
| EIF2S3-F | CCACAGTCGTCAAAGCTATTTCT |
| EIF2S3-R | CGAGGGCAACTTGGGTCATC |
| TRIO-F | AAACAGCTACACAGAGATTGGG |
| TRIO-R | ACACGTTCATACAGTTCATGGC |
| RCH3H1-F | TCCACAATGGACGGATTTCCT |
| RCH3H1-R | AACCCAAACTGATGGGCTTTC |
| APPL1-F | AGAGACCATGCAACAGACAATAG |
| APPL1-R | GTATCCAGCCTTTCGGGTTAAA |
