# Supplementary material for: tRF-3013b inhibits gallbladder cancer proliferation by targeting TPRG1L
Source: Cell Mol Biol Lett. 2022 Nov 18;27:99. doi: 10.1186/s11658-022-00398-6 (PMC9673407; doi:10.1186/s11658-022-00398-6)
Supplement: Supplementary file 2 — Additional file 2: Table S2. RNA oligos used in the study. [file 11658_2022_398_MOESM2_ESM.docx]

**Table S2. RNA oligos used in the study**

| **Name** | **sequence（5’-3’）** |
| --- | --- |
| BRF1-Si1 | GGACUGUGAAGGAGGUCAUTT |
| BRF1-Si2 | CCAGGAUGCAAUUGAGAUUTT |
| BRF1-Si3 | GCAUCUACAAGGAACACAATT |
| ANG-Si1 | UGUUGUUGGUCUUCGUGCUGGGUCU |
| ANG-Si2 | AGGAUAACUCCAGGUACACACACUU |
| ANG-Si3 | CCUGACCCAGCACUAUGAUGCCAAA |
| Dicer-Si1 | UUCAAUAUCCUCUUCUUUCUC |
| Dicer-Si2 | UCUUUUUCCCAUUUAUCUGUG |
| Dicer-Si3 | AAUAUUCUGCAAAAGUUUCAU |
| NC | UUCUCCGAACGUGUCACGUTT |
| Anti-NC | CAGUACUUUUGUGUAGUACAA |
| tRF-3013b mimic | UCGAAUCCGAGUCACGGCACCA |
| Anti- tRF-3013b | UGGUGCCGUGACUCGGAUUCGA |
| tRF-Gly-CCC mimic | GCAUUGGUGGUUCAGUGGUAGA |
