# Supplementary figures and images for: tRF-3013b inhibits gallbladder cancer proliferation by targeting TPRG1L
Source: Cell Mol Biol Lett. 2022 Nov 18;27:99. doi: 10.1186/s11658-022-00398-6 (PMC9673407; doi:10.1186/s11658-022-00398-6)

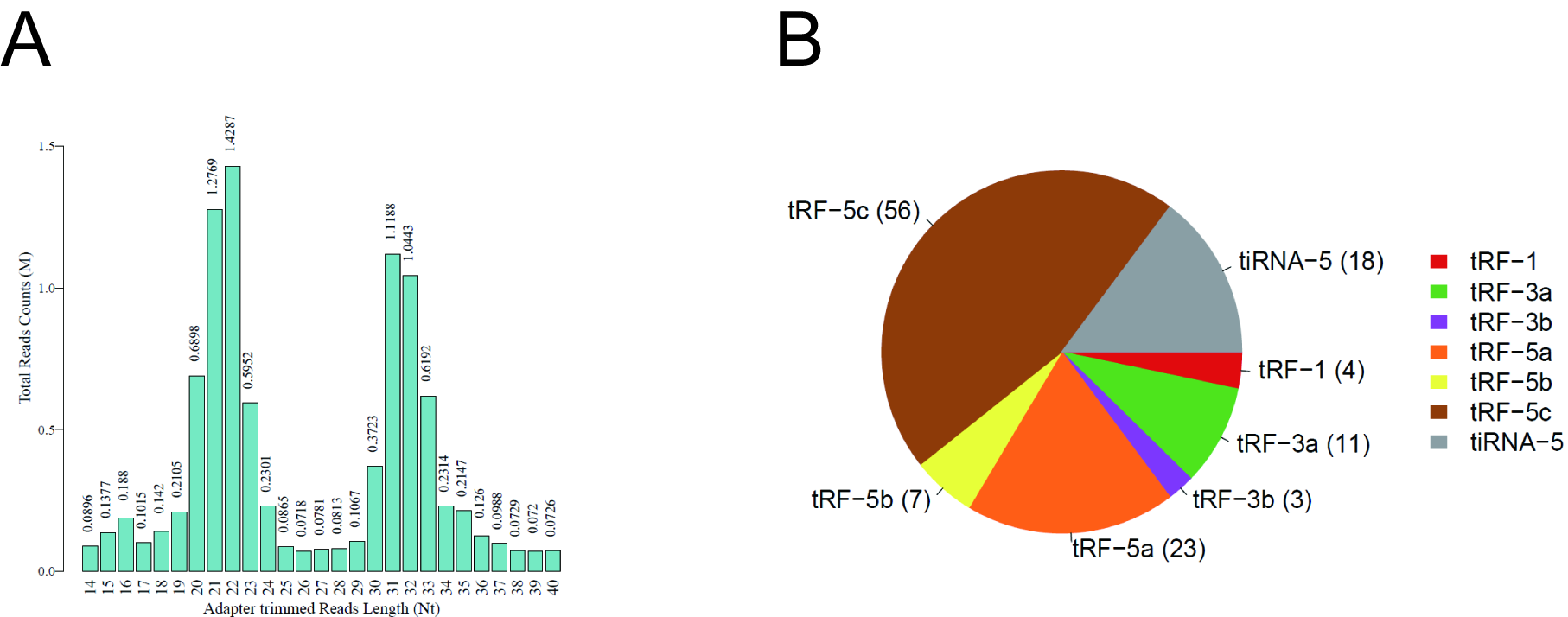

Supplement: Supplementary file 3 — Additional file 3: Fig S1. tRFs expression in GBC tissues. (A) Bar chart of the sequence read length distribution of tRF and tiRNA. (B) Pie chart of the distribution of subtype tRF and tiRNA. [file 11658_2022_398_MOESM3_ESM.tif]

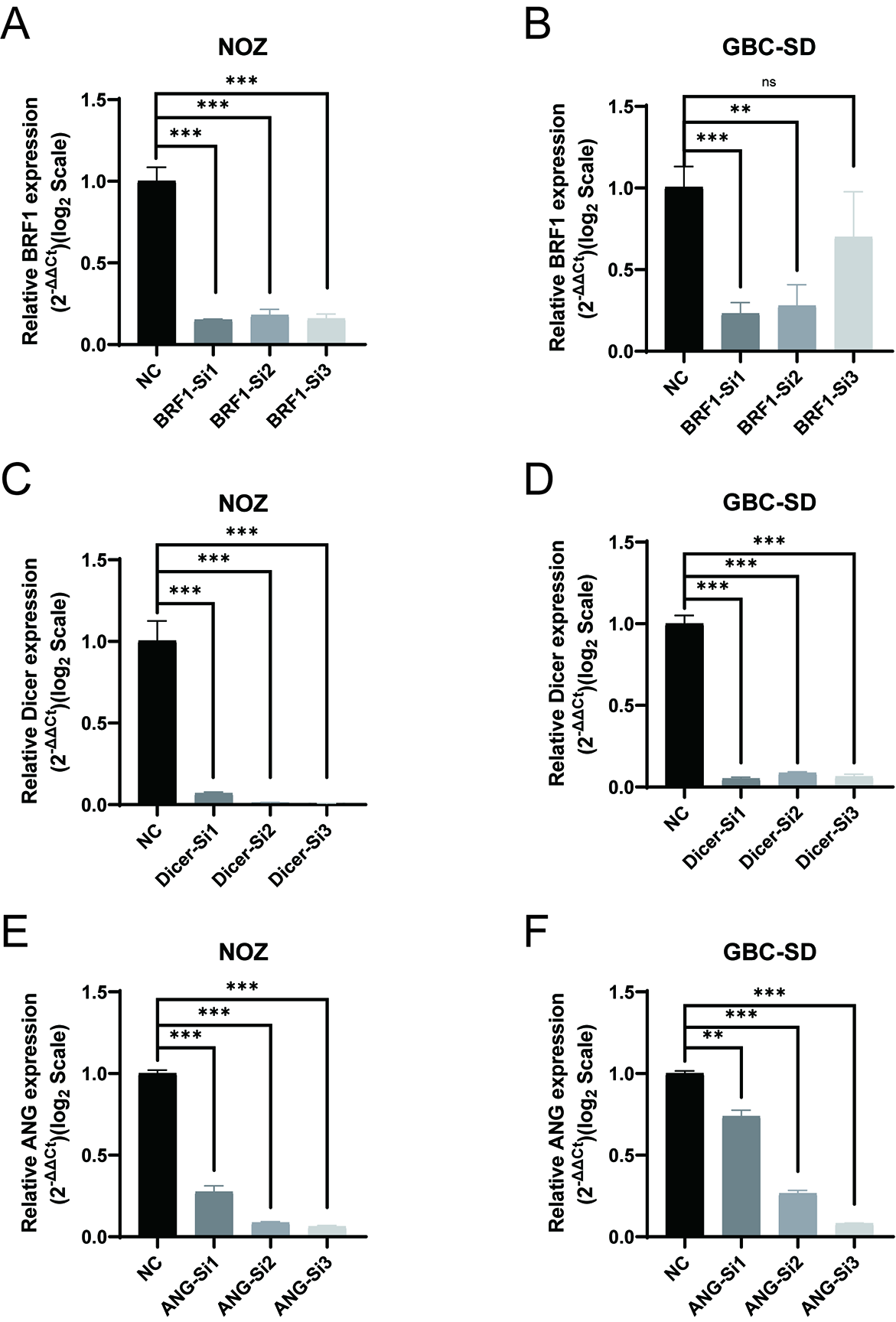

Supplement: Supplementary file 4 — Additional file 4: Fig S2. Verification of BRF1, Dicer, and ANG knockdown. (A, B) Relative mRNA expression of BRF1 in NOZ and GBC-SD cells with BRF1 knockdown. (C, D) Relative mRNA expression of Dicer in NOZ and GBC-SD cells with Dicer knockdown. (E, F) Relative mRNA expression of ANG in NOZ and GBC-SD cells with ANG knockdown. ns P > 0.05, **P < 0.01, ***P < 0.001. [file 11658_2022_398_MOESM4_ESM.tif]

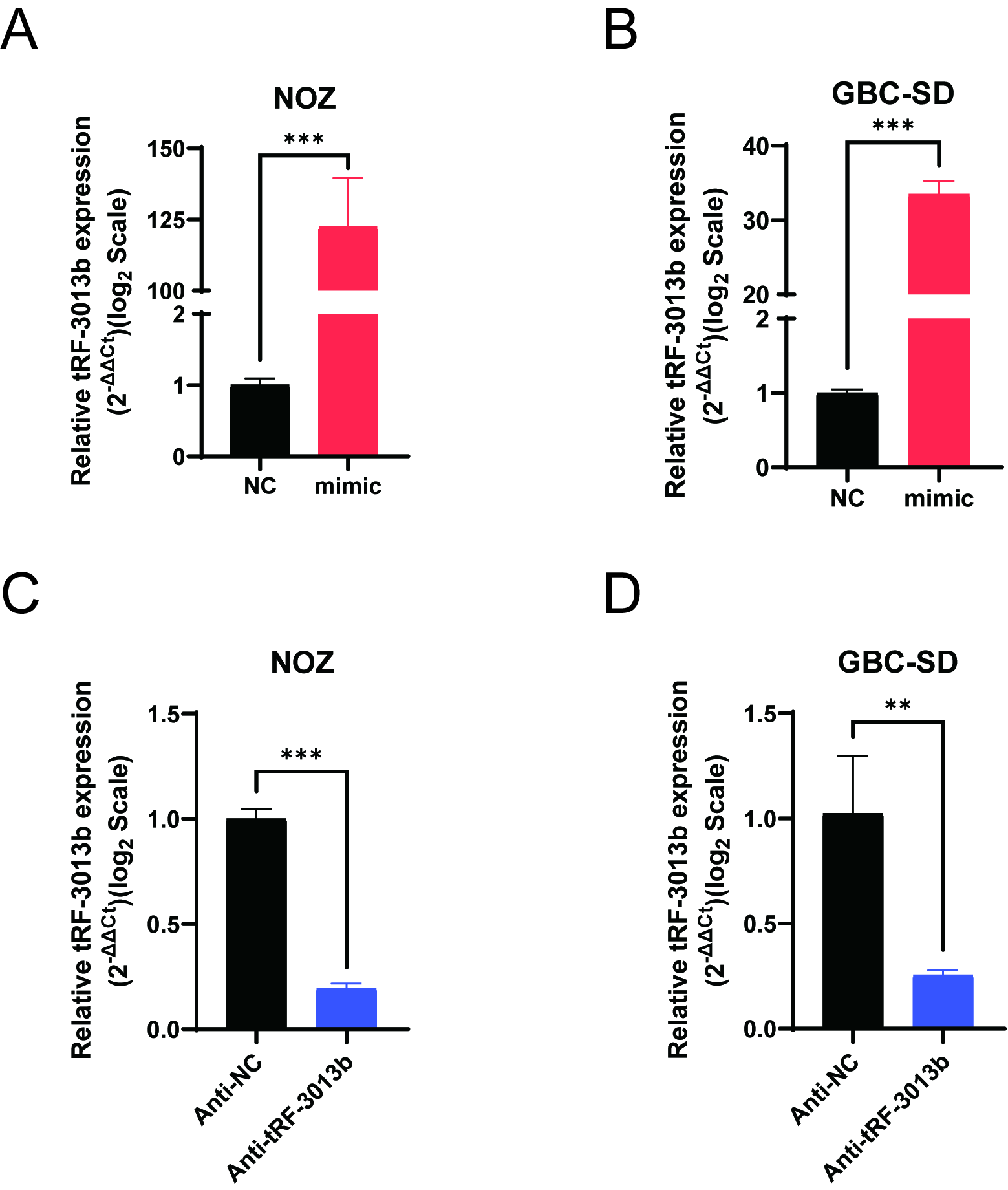

Supplement: Supplementary file 5 — Additional file 5: Fig S3. Verification of tRF-3013b overexpression and inhibition. (A, B) Relative tRF-3013b expression with NC or tRF-3013b mimic transfection in NOZ and GBC-SD cells. (C, D) Relative tRF-3013b expression with Anti-NC or Anti-tRF-3013b transfection in NOZ and GBC-SD cells. **P < 0.01, ***P < 0.001. [file 11658_2022_398_MOESM5_ESM.tif]

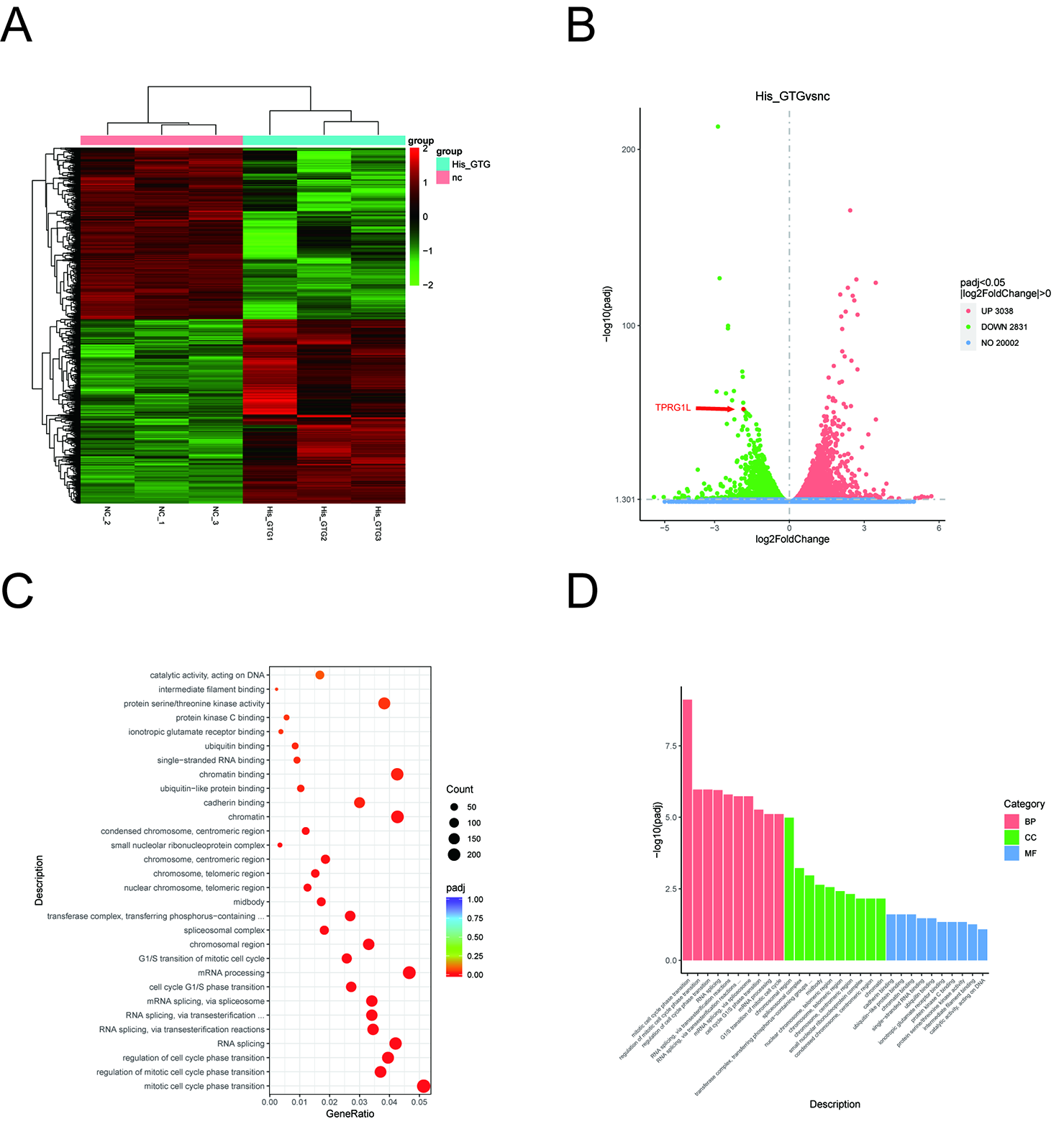

Supplement: Supplementary file 6 — Additional file 6: Fig S4. tRF-3013b was related to the cell cycle progression. (A) Heatmap showing the differential genes between NOZ cells transfected with NC or tRF-3013b mimic. (B) Volcano plot showing the differential genes between NOZ cells transfected with NC or tRF-3013b mimic. (C) GO enrichment distribution map showing the related pathways after transfection with NC or tRF-3013b mimic in NOZ cells. (D) Significance of related pathways by GO enrichment analysis (BP, biological process; CC, cellular component; MF, molecular function). [file 11658_2022_398_MOESM6_ESM.tif]

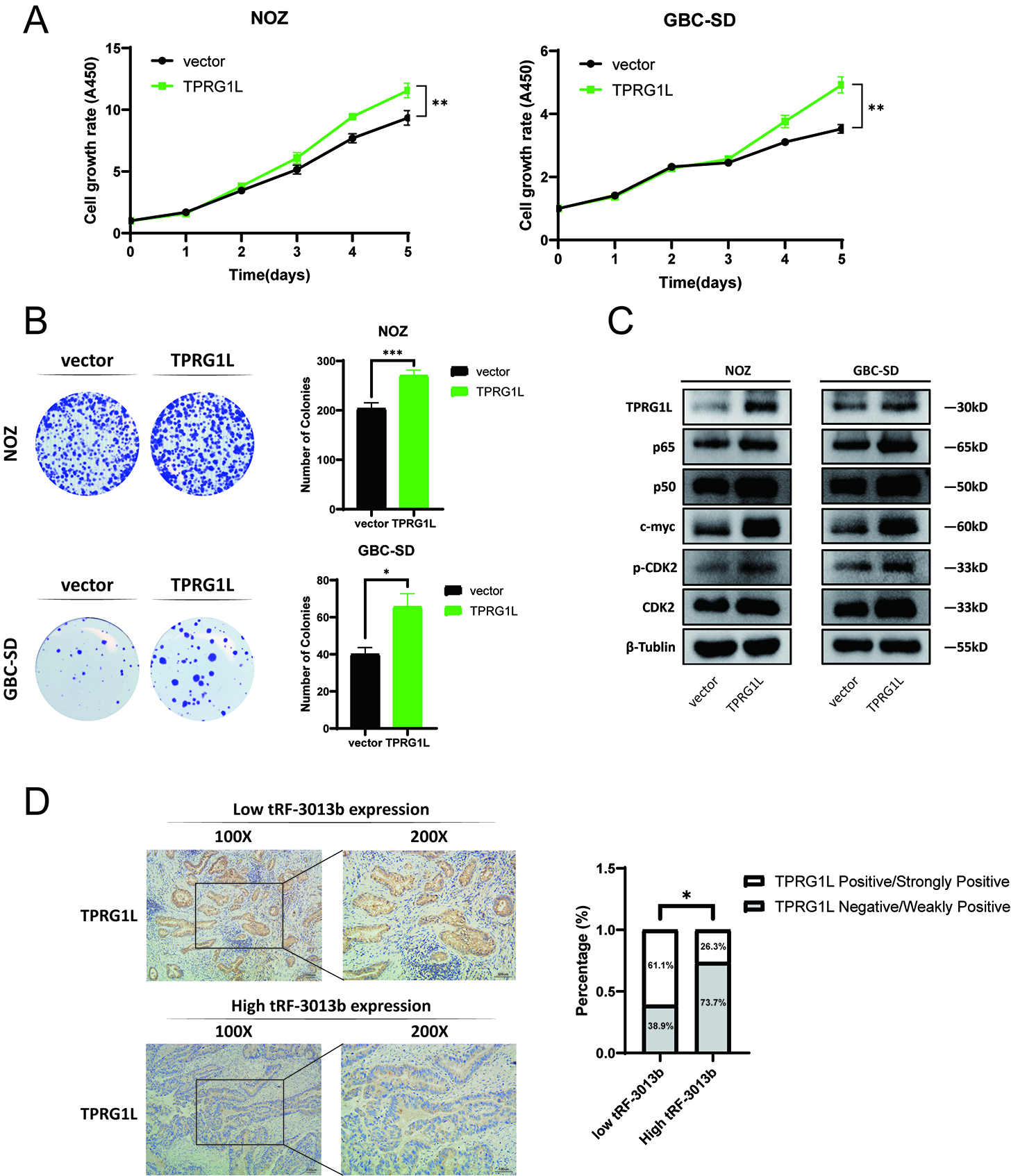

Supplement: Supplementary file 7 — Additional file 7: Fig S5. TPRG1L promoted GBC cell proliferation. (A) CCK8 assay and (B) colony formation were performed to evaluated proliferation capacity after transfection with vector or TPRG1L. (C) Relative protein expression of TPRG1L, p65, p50, c-myc, CDK2, and p-CDK2 after transfection with vector or TPRG1L. (D) Representative IHC micrographs showing TPRG1L protein expression in GBC tissues with high or low miR-143-3p expression. *P < 0.05, **P < 0.01, ***P < 0.001. [file 11658_2022_398_MOESM7_ESM.tif]
